# Health-care utilization for headache disorders in Nepal: a population-based door-to-door survey

**DOI:** 10.1186/s10194-018-0942-3

**Published:** 2018-11-28

**Authors:** Kedar Manandhar, Ajay Risal, Mattias Linde, Timothy J. Steiner

**Affiliations:** 1 Dhulikhel Hospital, Kathmandu University Hospital, Dhulikhel, Kavre Nepal; 2 Kathmandu University School of Medical Sciences, Dhulikhel, Kavre Nepal; 3 Department of Neuromedicine and Movement Science, Norwegian University of Science and Technology, Edvard Griegs Gate, NO-7489 Trondheim, Norway; 4 Norwegian Advisory Unit on Headache, St Olavs University Hospital, Trondheim, Norway; 5 Division of Brain Sciences, Imperial College London, London, UK

**Keywords:** Headache disorders, Health-care utilization, Medical consultation, Population-based study, Quality of care, Structured headache services, Nepal, Global campaign against headache

## Abstract

**Background:**

Headache disorders are an important global public-health problem, but under-diagnosed, undertreated and under-prioritized. Deficiencies in health care for headache, present everywhere, are likely to be greater in poorly-resourced countries. This study reports on health-care utilization for headache in Nepal, a low-income country with high headache burden.

**Methods:**

We took data from a cross-sectional, nationwide population-based door-to-door survey, with multistage cluster random sampling. Face-to-face structured interviews included enquiry into consultations with professional health-care providers (HCPs), and investigations and treatments for headache. Analysis included associations with sociodemographic variables and indices of symptom severity.

**Results:**

Of 2100 participants, 1794 reported headache during the preceding year (mean age 36.1 ± 12.6 years; male/female ratio 1:1.6). Of these, 58.4% (95% CI: 56.1–60.7%) had consulted at least once in the year with HCPs at any level, most commonly (25.0%) paramedical; 15.0% had consulted pharmacists, 10.8% general physicians and 7.6% specialists (of any type). Participants with probable medication-overuse headache consulted most (87.0%), followed by those with migraine (67.2%) and those with tension-type headache (48.6%; *p* < 0.001). A minority (11.9%) were investigated, mostly (8.9%) by eye tests. Half (50.8%) had used conventional medications for headache in the preceding month, paracetamol being by far the most common (38.0%), and 10.3% had used herbal therapies.

Consultation was positively associated with rural habitation (AOR = 1.5; *p* < 0.001). Proportions consulting increased in line with all indices of symptom severity.

**Conclusions:**

Although over half of participants with headache had consulted professional HCPs, this reflects demand, not quality of care. Although 7.6% had seen specialists, very few would have been headache specialists in any sense of this term. High persistent burden, with only half of participants with headache using conventional medications, and these not best chosen, suggests these consultations fell far short of meeting need. Health policy in Nepal should recognise this, since the consequences otherwise are costly: lost health, diminished productivity and damaged national economy. On a positive note, the proportions consulting suggest that capacity exists at multiple levels within the Nepalese health system. With this to build upon, structured headache services in line with international recommendations appear achievable in Nepal. Educational programmes are the essential requirement.

## Background

Headache disorders are among the most prevalent, burdensome and costly diseases in the world [1–3]. From a public-health perspective, the primary headache disorders, mostly migraine and tension-type headache (TTH), are of special importance because they lead to widespread ill health, impaired quality of life and much loss of productivity [3]. Inappropriate management of either migraine or TTH can lead to medication-overuse headache (MOH), a major additional contributor to global disability [4].

The Global Burden of Disease Study 2015 (GBD2015) ranked TTH and migraine as second and fifth most prevalent disorders worldwide [5]. In GBD2016, migraine was the second highest cause of disability [6]. These disorders are not the preserve of wealthy countries: recent epidemiological studies in low- and middle-income (LAMI) countries (Ethiopia [7, 8], India [9, 10], Pakistan [11] and Zambia [12, 13]) all found higher prevalence estimates of migraine than the global mean, with substantial headache-attributed burden at individual and population levels. Despite these findings, headache disorders are under diagnosed, undertreated and under-prioritized in health-care delivery systems, and this is especially so in LAMI countries [1].

Nepal is one of the poorest countries within the South-East Asia Region (SEAR). Of its population of approximately 30 million, about one quarter live below the international poverty line [14]. In a population-based study in this country, we found an exceptionally high 1-year prevalence of migraine (34.1%), while the prevalence of probable MOH (pMOH) (2.1%) was towards the upper end of the range observed in other countries [15]. Headache-attributed burden was accordingly high: migraine, TTH and pMOH were accountable for reduced functional capacities of 0.81%, 0.06% and 0.20% respectively at population level [16].

Extreme geographical variation in Nepal causes difficulties of access, aggravated by poor infrastructure such as bad roads and rickety bridges, and a monsoon climate [17], while limited education generally, lack of skill in headache disorders among health professionals, and low investment in health care are high barriers to effective headache care in Nepal. This study assesses health-care utilization for headache and its associations with sociodemographic variables and indices of symptom severity in Nepal using data from the same nationwide population-based survey. The purpose was to provide evidence for national health policy and formulation of public-health programmes in Nepal.

## Methods

### Study design

The detailed methods have been described elsewhere [17, 18]. In summary, this was a cross-sectional, nationwide population-based door-to-door survey. Trained interviewers made unannounced visits to households selected through stratified multistage cluster random sampling, and conducted face-to-face structured interviews with one adult randomly selected from each household. Representativeness was achieved by sampling in all three physiographic divisions (Mountain, Hill and Terai), and, within each division, all five development regions (Far-western, Mid-western, Western, Central and Eastern). In total, 2100 Nepali-speaking adults aged 18–65 years and resident in Nepal were included during May 2013.

### Instrument

Interviewers used the Headache-Attributed Restriction, Disability, Social Handicap and Impaired Participation (HARDSHIP) modular structured questionnaire developed by *Lifting The Burden* (LTB) for population-based studies [19]. This was culturally adapted, and translated into Nepali language according to LTB’s translation protocol for hybrid documents [20]. It included multiple elements. First were (i) demographic enquiry and (ii) a neutral headache screening question (“Have you had a headache during the last 12 months?”) addressed to all participants. Then, for those who screened positively, were (iii) diagnostic questions based on the International Classification of Headache Disorders (ICHD) [21] (focused on the subjectively most bothersome type in those reporting more than one), and (iv) questions regarding various aspects of headache-attributed burden. The last included indices of symptom severity: headache frequency (days/month [d/m]), attack duration (hours) and headache intensity (with response options “not bad”, “quite bad” and “very bad”, which we interpreted as mild, moderate and severe).

Last, of those with headache, we enquired about (v) consultations for headache (yes or no, and with whom: see below), (vi) investigations for headache undergone within the preceding year (particularly specifying xrays of paranasal sinuses [PNS] or neck, brain imaging [CT or MRI], EEG and eye tests), and (vii) treatments for headache (conventional medications and/or herbal preparations) in the preceding month.

### Headache diagnosis

The diagnostic method, centrally by algorithm, has been described previously [15]. Participants reporting headache on ≥15 d/m were first separated; those also overusing acute medication were considered to have pMOH [22] while the remainder were categorized as “other headache on ≥15 d/m” (these cannot be fully diagnosed by questionnaire). To all others, reporting headache on ≤14 d/m, the algorithm applied modified ICHD-3 beta criteria [21] in the following order: definite migraine, definite TTH, probable migraine and probable TTH. Definite and probable migraine were combined, and likewise definite and probable TTH, for further analyses. The few remaining cases were unclassifiable.

### Medical consultation

We defined “medical consultation” operationally as consultation, at least once in the preceding year, with any professional health-care provider (HCP) by a participant reporting any type of headache. We classified HCPs into four groups: (i) pharmacist; (ii) paramedic (nurse, physiotherapist, health assistant, auxiliary health worker or herbal practitioner); (iii) general physician; or (iv) specialist (headache specialist, neurologist, neurosurgeon, ophthalmologist, ear, nose and throat [ENT] specialist or psychiatrist). We ranked these groups in ascending order, (i) to (iv).

### Statistical analyses

We counted participants with headache reporting medical consultation(s), investigations and/or treatments, and calculated proportions (%) with 95% confidence intervals (CIs). Those reporting consultations at multiple levels were counted only within the highest-ranked group.

We analysed demographic variables as follows. We categorized age in years into five groups: 18–25, 26–35, 36–45, 46–55, 56–65. We used household consumption in USD/year (at the time of study: 1 USD ≈ NPR 100) as an indicator of economic wellbeing, categorizing it in three groups: poorest (< 950), poor (950–1200) and intermediate and above (> 1200). We classed habitation as urban or rural, and dichotomized altitude of dwelling into < 1000 m and ≥ 1000 m.

As indices of symptom severity, we categorized headache frequency in d/m (F) into four groups (< 1, 1–2, 3–14, ≥15) and attack duration in hours (D) into three (< 4, 4–12, > 12). Because F reflected days with headache rather than attack frequency, we used a conservative approximation method for calculation of proportion of time in ictal state (pT). For participants reporting D ≤ 24 h, pT was calculated as [(F*D)/(30*24)]. When D > 24 h, we assumed F was accordingly inflated (for example, one attack lasting into 2 days was reported as 2 headache days); we therefore calculated pT as [(F*24)/(30*24)] = F/30. We converted proportions into percentages, and categorized these into four groups (< 1%, 1–2%, 2.1–8%, > 8%).

We used chi-squared to test differences in proportions reporting medical consultation(s), investigations and/or treatments, according to headache type, habitation and/or dwelling altitude. We used bivariate and multivariate logistic regression analyses (with odds ratios [ORs] and adjusted ORs [AORs], each with 95% CIs) to investigate associations of medical consultation with demographic variables and indices of symptom severity. We entered each of these as covariates in the multivariate analyses, but only the demographic variables for associations with symptom severity. We considered *p* < 0.05 to be statistically significant.

We analysed all data using SPSS 21.0 (IBM Corp, Armonk, NY, USA).

## Results

A total of 2100 participants were included in the original survey, but here we analyze only the 1794 who reported headache during the preceding year (mean age 36.1 ± SD 12.6 years; M/F ratio 1:1.6; 38.2% from poorest category; 38.2% rural; 52.9% dwelling at altitude ≥1000 m).

### Medical consultation

**Fig. 1:**
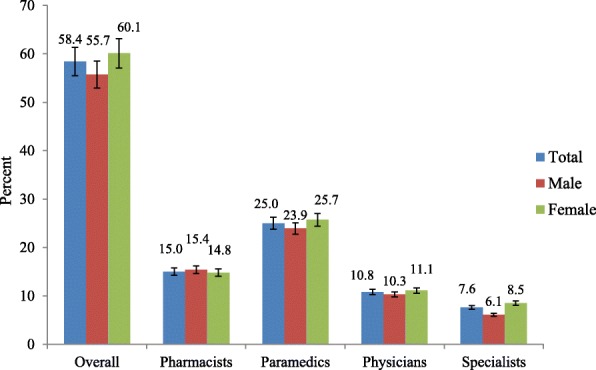
Medical consultation according to class of professional health-care provider, by gender (proportions, with 95% confidence intervals)

Of the 1794 participants reporting headache, 1048 (58.4%; 95% CI: 56.1–60.7%) consulted at some level within the preceding year for headache, 25.0% (M/F ratio 1:1.1) with paramedics, 15.0% (M/F ratio 1:0.96]) with pharmacists, 10.8% (M/F ratio 1:1.1) with general physicians and 7.6% (M/F ratio: 1:1.4) with specialists (Fig. 1). By headache type, participants with pMOH (87.0%) were most likely to have consulted, followed by those with migraine (67.2%), then those with TTH (48.6%; *p* < 0.001) (Table 1).

**Table 1:** Medical consultation according to class of professional health-care provider, by headache type

Headache type	Medical consultation					
	N	Pharmacist	Paramedic	Physician	Specialist	Overall
		n (%) [95% confidence interval]				
Tension-type headache	863	123 (14.3) [12.0–16.8]	185 (21.4) [18.7–24.3]	64 (7.4) [5.8–9.4]	47 (5.4) [4.0–7.2]	419 (48.6) [45.2–51.9]
Migraine	728	117 (16.1) [13.5–18.9]	216 (29.7) [26.4–33.1]	101 (13.9) [11.4–16.6]	55 (7.6) [5.7–9.7]	489 (67.2) [63.6–70.6]
Probable medication-overuse headache	46	9 (19.6) [9.4–33.9]	10 (21.7) [10.9–36.4]	11 (23.9) [12.6–38.8]	10 (21.7) [10.9–36.4]	40 (87.0) [73.7–95.6]
p*	–	< 0.001	< 0.001	< 0.001	< 0.001	< 0.001

p*: chi-squared test

### Investigations and treatments

Of the 1794 participants reporting headache, rather more than one tenth (11.9%) were investigated for headache within the preceding year, most commonly (8.9%) by eye test (Table 2). Next, but far fewer, were xrays of PNS (1.4%); brain imaging (0.9%) was unusual, and neck xrays (0.4%) and EEG (0.3%) even more so (Table 2).

**Table 2:** Investigations and treatments for headache of participants with any headache, by habitation and dwelling altitude

	All	Habitation			Dwelling altitude		
		Urban	Rural	p*	< 1000 m	≥1000 m	p*
	n (%) [95% CI]				n (%) [95% CI]		
Investigations in the preceding year							
All	214 (11.9) [10.5–13.5]	75 (10.9) [8.7–13.5]	139 (12.5) [10.7–14.6]	0.33	106 (12.5) [10.4–15.0]	108 (11.4) [9.4–13.6]	0.47
Xray of paranasal sinuses	26 (1.4) [0.9–2.1]	5 (0.7) [0.2–1.7]	21 (1.9) [1.2–2.9]	0.07	12 (1.4) [0.7–2.5]	14 (1.5) [0.8–2.5]	0.99
Xray of neck	7 (0.4) [0.2–0.6]	3 (0.4) [0.1–1.3]	4 (0.4) [0.1–0.9]	1.0	1 (0.1) [0.0–0.7]	6 (0.6) [0.2–1.4]	0.13
Brain imaging (CT or MRI)	16 (0.9) [0.5–0.9]	7 (1.0) [0.4–2.1]	9 (0.8) [0.4–1.5]	0.79	9 (1.1) [0.5–2.0]	7 (0.7) [0.3–1.5]	0.61
EEG	5 (0.3) [0.1–0.5]	1 (0.1) [0.0–0.8]	4 (0.4) [0.1–0.9]	0.65	0 (0.0) -	5 (0.5) [0.2–1.2]	–
Eye test	160 (8.9) [7.6–10.3]	59 (8.6) [6.6–11.0]	101 (9.1) [7.5–11.0]	0.73	82 (9.7) [7.8–11.9]	78 (8.2) [6.6–10.2]	0.28
Conventional medications in the preceding month							
Overall	912 (50.8) [48.5–53.2]	299 (43.6) [39.8–47.4]	613 (55.3) [52.3–58.3]	**< 0.001**	393 (46.5) [43.1–49.9]	519 (54.7) [51.5–57.9]	**< 0.001**
Paracetamol	682 (38.0) [35.8–40.3]	203 (29.6) [26.2–33.2]	479 (43.2) [41.9–47.8]	**< 0.001**	250 (29.6) [26.5–32.8]	432 (45.5) [42.3–48.8]	**< 0.001**
Ibuprofen	150 (8.4) [7.1–9.7]	57 (8.3) [6.4–10.6]	93 (8.4) [6.8–10.2]	0.99	51 (5.9) [4.5–7.9]	99 (10.4) [8.6–12.6]	**0.001**
Nimesulide	94 (5.2) [4.3–6.4]	49 (7.1) [5.3–9.3]	45 (4.1) [3.0–5.4]	**0.009**	68 (8.0) [6.3–10.1]	26 (2.7) [1.8–4.0]	**< 0.001**
NSAID/paracetamol combination	71 (4.0) [3.1–5.0]	18 (2.6) [1.6–4.1]	53 (4.8) [3.6–6.2]	**0.025**	43 (5.1) [3.7–6.8]	28 (3.0) [2.0–4.1]	**0.021**
Herbal therapies in the preceding month							
Overall	185 (10.3) [8.9–11.8]	36 (5.2) [3.7–7.2]	149 (13.4) [11.5–15.6]	**< 0.001**	63 (7.5) [5.8–9.4]	122 (12.9) [10.8–15.2]	**< 0.001**
Vicks	64 (3.6) [2.8–4.6]	15 (2.2) [1.2–3.6]	49 (4.4) [3.3–5.8]	**0.013**	36 (4.3) [3.0–5.8]	28 (3.0) [3.0–4.3]	0.16
Tulsi leaves	11 (0.6) [0.3–1.1]	3 (0.4) [0.1–1.3]	8 (0.7) (0.3–1.4)	0.55	7 (0.8) [0.3–1.7]	4 (0.4) [0.1–1.1]	0.37
Titepati leaves	11 (0.6) [0.3–1.1]	3 (0.4) [0.1–1.3]	8 (0.7) [0.3–1.4]	0.55	2 (0.2) [0.0–0.9]	9 (0.9) [0.4–1.8]	0.069

*CI* Confidence interval

p*: chi-squared test

Significant p-values (<0.05) are emboldened

Just over half (50.8%) had used at least one type of conventional medication for headache in the preceding month. Among 13 different medications (paracetamol, aspirin, diclofenac, ibuprofen, naproxen, nimesulide, codeine or dihydrocodeine, domperidone, metoclopramide, Codamol, Coflam, D-cold and Rhinex), the most common was paracetamol as monotherapy (38.0%); ibuprofen (8.4%) followed way behind. Nimesulide was used by a not inconsiderable minority (5.2%). Less common was the combination of nonsteroidal anti-inflammatory drug (NSAID) and paracetamol (4.0%) (Table 2).

Just over one tenth (10.3%) had used herbal therapies in the last month, most commonly Vicks (3.6%). This was usually applied to the forehead, inhaled or administered via the nasal mucosa. Depending on formulation, it might contain camphor, turpentine oil, levomenthol, eucalyptus oil, Siberian pine needle oil, methyl salicylate and/or oxymetazoline hydrochloride. Next, but not common, were tulsi leaves (Holy basil, or *Ocimum tenuiflorum*) and titepati leaves (*Artemesia vulgaris*) (both 0.6%) (Table 2). These were usually taken as a drink after boiling in water.

### Associations

Overall, medical consultations were similar in females (60.1%) and males (55.7%; *p* = 0.96) (Fig. 1, Tables 3 and 4). Females (8.5%) consulted specialists more than males (6.1%), but not significantly (*p* = 0.28) (Tables 3 and 4).

**Table 3:** Medical consultation by age, gender, household consumption, habitation and dwelling altitude

Variable	N	Overall		Specialist	
		n (%)	95% CI	n (%)	95% CI
Age (years)					
18–25	426	240 (56.3)	51.5–61.1	24 (5.6)	3.6–8.3
26–35	581	346 (59.6)	55.4–63.6	54 (9.3)	7.1–12.0
36–45	358	204 (57.0)	51.7–62.2	25 (7.0)	4.6–10.1
46–55	258	162 (62.9)	56.6–68.7	18 (7.0)	4.2–10.8
56–65	171	96 (56.1)	48.4–63.7	15 (8.8)	5.0–14.1
Gender					
Male	689	384 (55.7)	51.9–59.5	42 (6.1)	4.4–8.2
Female	1105	664 (60.1)	57.1–63.0	94 (8.5)	6.9–10.3
Household consumption (USD/year)					
< 950	686	423 (61.7)	57.9–65.3	46 (6.7)	5.0–8.8
950–1200	687	400 (58.2)	54.4–61.9	61 (8.9)	6.9–11.3
> 1200	421	225 (53.4)	48.6–58.3	29 (6.9)	4.7–9.7
Habitation					
Urban	686	354 (51.6)	47.8–55.4	54 (7.9)	6.0–10.1
Rural	1108	694 (62.6)	59.7–65.5	82 (7.4)	5.9–9.1
Dwelling altitude (m)					
< 1000	845	474 (56.1)	52.7–59.5	72 (8.5)	6.7–10.6
≥ 1000	949	574 (60.5)	57.3–63.6	64 (6.7)	5.2–8.5

*CI* confidence interval

**Table 4:** Associations between medical consultation and demographic variables

Variable	Overall				Specialist consultation			
	Bivariate analysis		Multivariate analysis		Bivariate analysis		Multivariate analysis	
	OR (95% CI)	p	AOR ^a^ (95% CI)	p	OR (95% CI)	p	AOR ^a^ (95% CI)	p
Age (in years)								
18–25	Reference	–	Reference	–	Reference	–	Reference	–
26–35	1.1 (0.9–1.5)	0.31	1.1 (0.8–1.4)	0.54	1.7 (1.1–2.8)	**0.034**	1.5 (0.9–2.5)	0.11
36–45	1.0 (0.8–1.4)	0.86	0.9 (0.7–1.3)	0.62	1.3 (0.7–2.4)	0.44	1.2 (0.6–2.1)	0.65
46–55	1.3 (0.6–1.8)	0.10	1.2 (0.9–1.7)	0.28	1.3 (0.7–2.4)	0.48	1.1 (0.6–2.2)	0.71
56–65	1.0 (0.7–1.5)	0.96	0.8 (0.6–1.2)	0.28	1.6 (0.8–3.2)	0.16	1.5 (0.8–3.0)	0.24
Gender								
Male	Reference	–	Reference	–	Reference	–	Reference	–
Female	1.2 (0.9–1.5)	0.069	1.0 (0.8–1.2)	0.96	1.4 (1.0–2.1)	0.062	1.2 (0.8–1.8)	0.28
Household consumption (USD/year)								
< 950	1.2 (0.9–1.4)	0.19	1.1 (0.9–1.4)	0.40	0.7 (0.5–1.1)	0.13	0.8 (0.5–1.1)	0.17
950–1200	Reference	–	Reference	–	Reference	–	Reference	–
> 1200	0.8 (0.6–1.1)	0.12	0.8 (0.6–1.0)	0.11	0.8 (0.5–1.2)	0.24	0.7 (0.5–1.2)	0.20
Habitation								
Urban	Reference	–	Reference	–	Reference	–	Reference	–
Rural	1.6 (1.3–1.9)	**< 0.001**	1.5 (1.2–1.9)	**< 0.001**	0.9 (0.7–1.3)	0.71	0.9 (0.6–1.3)	0.51
Dwelling altitude(m)								
< 1000	Reference	–	Reference	–	Reference	–	Reference	–
≥ 1000	1.2 (0.9–1.4)	0.06	1.0 (0.8–1.2)	0.96	0.8 (0.6–1.1)	0.16	0.7 (0.5–1.1)	0.094

*OR* odds ratio, *CI* confidence interval, *AOR* adjusted OR

^a^ adjusted for age, gender, household consumption, habitation, dwelling altitude, headache frequency, attack duration, headache intensity and proportion of time in ictal state

Significant p-values (<0.05) are emboldened

There was a slightly increasing overall trend with advancing age up to 46–55 years (from 56.3% for 18–25 years to 62.9% for 46–55 year), then a decrease (to 56.1% for 56–65 years) (Tables 3 and 4). However, age was not a significant factor (AOR 1.2 [*p* = 0.28] for 46–55 years and AOR 0.8 [*p* = 0.28] for 56–65 years with reference to the youngest group) (Tables 3 and 4). Consultations with specialists increased with age (5.6% for 18–25 years, 9.3% for 26–35 years, 7.0% for 36–55 years and 8.8% for 56–65 years), but again not significantly (Tables 3 and 4).

Medical consultation showed no associations with household consumption or dwelling altitude. Rural participants consulted more than urban (OR = 1.6; AOR = 1.5; *p* < 0.001) (Tables 3 and 4). Specialist consultation showed no demographic associations.

Investigations showed no associations, but both conventional medications and herbal therapies were reportedly used more, overall, by rural than by urban participants (*p* < 0.001), and by high-altitude dwellers than low (*p* < 0.001) (Table 2).

Associations with indices of symptom severity are presented in Table 5. In bivariate analysis, greater severity (according to all indices) was associated with increased likelihood of medical consultation. Multivariate analysis confirmed these associations. This was not the case for specialist consultation. Greater headache frequency showed a paradoxical association, with ORs and AORs of 0.6–0.7 for frequencies of 1–14 d/m but > 2 for frequency ≥ 15 d/m. Reflecting this, specialist consultation was less likely initially (OR and AOR = 0.5) as pT increased above 2%, then becoming more likely beyond pT = 8%. Greater headache intensity was clearly associated with specialist consultation in both analyses, whereas the effect of attack duration did not achieve significance (Table 5).

**Table 5:** Associations between medical consultation and indices of symptom severity

Index	Overall				Specialist consultation			
	Bivariate analysis		Multivariate analysis		Bivariate analysis		Multivariate analysis	
	OR (95% CI)	p	AOR ^a^ (95% CI)	p	OR (95% CI)	p	AOR ^a^ (95% CI)	p
Headache frequency (d/m)								
< 1	Reference	–	Reference	–	Reference	–	Reference	–
1–2	1.2 (0.9–1.5)	0.18	1.2 (0.9–1.5)	0.17	0.6 (0.4–0.9)	**0.025**	0.6 (0.4–0.9)	**0.023**
3–14	1.8 (1.4–2.3)	**< 0.001**	1.9 (1.5–2.4)	**< 0.001**	0.7 (0.4–1.1)	0.14	0.7 (0.4–1.1)	0.12
≥ 15	2.7 (1.8–3.9)	**< 0.001**	2.6 (1.8–3.9)	**< 0.001**	2.2 (1.3–3.6)	**0.002**	2.1 (1.3–3.5)	**0.004**
Attack duration (hours)								
< 4	Reference	–	Reference	–	Reference	–	Reference	–
4–12	2.0 (1.6–2.6)	**< 0.001**	2.1 (1.6–2.6)	**< 0.001**	1.5 (0.9–2.3)	0.13	1.4 (0.9–2.3)	0.14
> 12	3.1 (2.5–4.3)	**< 0.001**	3.3 (2.3–4.1)	**< 0.001**	1.5 (0.9–2.2)	0.087	1.4 (0.9–2.4)	0.10
Headache intensity								
Mild	Reference	–	Reference	–	Reference	–	Reference	–
Moderate	2.5 (2.0–3.2)	**< 0.001**	2.5 (2.0–3.2)	**< 0.001**	1.3 (0.8–2.3)	0.30	1.3 (0.8–2.3)	0.31
Severe	3.8 (2.9–5.1)	**< 0.001**	3.6 (2.7–4.8)	**< 0.001**	2.6 (1.5–4.5)	**0.001**	2.7 (1.3–4.7)	**< 0.001**
Proportion of time in ictal state (pT) (%)								
< 1	Reference	–	Reference	–	Reference	–	Reference	–
1–2	1.5 (1.1–2.0)	**0.012**	1.5 (1.1–2.0)	**0.014**	1.0 (0.6–1.7)	0.92	1.0 (0.5–1.7)	0.95
2.1–8	1.6 (1.2–2.1)	**0.001**	1.6 (1.2–2.1)	**0.001**	0.5 (0.2–0.9)	**0.023**	0.5 (0.3–0.9)	**0.021**
> 8	2.8 (2.2–3.5)	**< 0.001**	2.9 (2.3–3.7)	**< 0.001**	1.4 (0.9–2.1)	0.094	1.4 (0.9–2.0)	0.13

*OR* odds ratio, *CI* confidence interval, *AOR* adjusted OR

^a^ adjusted for age, gender, household consumption, habitation and dwelling altitude

Significant p-values (<0.05) are emboldened

## Discussion

We found that almost three fifths (58.4%) of all participants reporting headache had at least one consultation with at least one class of professional HCP in the previous year, while fewer than one-tenth (7.6%) had seen a specialist of any sort. Unsurprisingly, the great majority (87.0%) of participants with pMOH had consulted, as had two-thirds (67.2%) with migraine but barely half (48.7%) with TTH. In keeping with this, the likelihood of having consulted increased with all indices of symptom severity, although only headache intensity and frequency of ≥15 d/m showed clear associations with specialist consultation. Over one tenth had been investigated. Half had used conventional medications, and one tenth had used herbal therapies for headache in the preceding month.

It may seem remarkable that such a large proportion of the participants with headache had consulted: this finding, in a low-income country, suggests better availability of health care than in many other, wealthier countries in Asia [23, 24], Western Europe [25–27] and North America [28, 29]. We need to make two cautionary comments on this. First, the evidence from other countries came from studies conducted some two decades earlier [24–29]. In that period, more effective treatments for headache have emerged, awareness of headache as a major public-health concern has substantially increased [30, 31], and, of course, information technology has advanced greatly. These may have led to changes in policy priorities and in public health-seeking behaviour. Second, and probably more influential, we included consultations with a very wide range of HCPs in our count of “medical consultations” in Nepal, among them some who have no counterparts in many other countries, or would not be accredited as health professionals. This probably lies behind the otherwise surprising finding that medical consultation was positively associated with rural habitation (discussed below). If pharmacist consultations (15.0%) are excluded, as they might be in studies elsewhere, the medical consultation proportion falls to 43.4% in Nepal, similar to the 46.6% recently found in China [32] and somewhat lower than findings in other countries [23–29]. If consultations only with physicians (general physicians [10.8%] or specialists [7.6%]) are considered, the proportion in Nepal (18.4%) is much lower than those elsewhere [23–29, 32]. This is probably the most salient comparison. It should be added that, while we included a range of specialties in the “specialist” classification (allowing for the possibility of consultations abroad), Nepal has no headache specialists, and few neurologists or neurosurgeons, so consultations were most likely with ophthalmologists, ENT specialists or psychiatrists.

In other words, these findings should not be taken as indicators of good care. The high consultation proportion reflects high demand, but gives no assurance that needs were met. On the contrary, headache-attributed burden in Nepal persists at a high level [16]. Investigations were requested for over 10%, but most were eye (vision) tests. While these might have been clinically indicated, they would not have contributed much to headache diagnosis or management [21]. Among the medications used, paracetamol was prominent – far more than NSAIDs, despite being less effective [33]. Nimesulide, a drug giving rise to serious safety concerns [34], was reportedly used by over 5%. These are clear markers of health-care failure.

But a more positive view may be taken. Over half of participants with headache had engaged at some level with professional health care, a proportion well in line with international recommendations for headache service organisation and delivery [31]. This indicates that capacity is available within Nepalese health services, and this can be built upon in a programme aimed at improvement. Furthermore, there is an existing health-care infrastructure that would readily accommodate the suggested three-tier model of headache-service organization [31]. Consultations with pharmacists (whose important role is to keep those with simple requirements outside the three-tier model) might be entirely adequate for the 15.0% reporting only these – if pharmacists had basic knowledge and skills in management of headache. This proviso applies equally to nurses, health assistants and auxiliary health workers (who would be at level one within the model) and, indeed, general physicians (level two). While only 10.8% had seen general physicians, and only 7.6% had seen a specialist of any sort (level three), these should suffice if all HCPs received training in headache care appropriate to their level [31].

Nepal is a low-income country; the range of household consumption (a marker of socio-economic status) is very much compressed towards the lower end. We did not see associations between household consumption and medical consultation, overall or with specialists, but any such relationship would be difficult to detect for this reason. We did observe that rural participants with headache were more likely than their urban counterparts to consult, although not with specialists. This, seen also in both Taiwan [24] and mainland China [32], may be due in Nepal to the provision nationwide but especially in rural areas of easy-access community-based primary health-care centres (PHCs), staffed mainly by health assistants and/or auxiliary health workers [35]. Nepal has a mixed health-service model, involving both public and private sectors. While government-supported basic services are available throughout the country, PHCs offer free medical consultations as well as being easily accessible [35]. Private-sector health care, predominantly available in urban areas, requires payment. Other likely factors are greater rural recourse to herbal practitioners, while a lesser tendency to self-medication in rural areas may drive people to consult health professionals.

As anticipated, all indices of symptom severity were positive predictors of medical consultation. Only headache intensity and high frequency predicted consultation with specialists, but numbers in these analyses were small, while attack duration (also a factor in proportion of time in ictal state) tends to be unreliably reported [19, 22]. A point worthy of emphasis here is that these indices of symptom severity are, clearly, key indicators of need for headache services. We have previously shown that all symptom indices increase with altitude of dwelling across the range < 500 m to 2499 m [36], and this was reflected here in the greater use of medication by high-altitude dwellers. Clearly, this relative excess of need among high-altitude dwellers presents a major challenge to equitable provision of headache services in Nepal. But need is very high in Nepal [15, 16], so it needs to be done.

### Strengths and limitations

The cross-sectional study from which our data were drawn used methods tested earlier in many countries, including India with a not dissimilar culture [9, 10]. Despite major logistic difficulties [15], it recruited a large, nationally representative sample through careful random selection, and minimized participation bias by achieving a participation proportion of > 99% [18]. These were strengths of the study.

Two limitations were inherent in population-based surveys. Enquiry into medical consultations was based on participants’ recall over the preceding year, with some degree of error expected. We were similarly dependent on participants’ truthfulness, although they had no reason to be evasive in this type of enquiry. Any errors that were introduced were more likely to be random than systematic [32, 37]. A third limitation was that no enquiry was possible into outcomes – in particular, satisfaction with care received as a result of consultation. This would have required much more detailed enquiry, beyond our resources. What is clear, since the burden of headache remains very high [16], is that needs in Nepal are largely unmet, despite the high proportion consulting HCPs.

## Conclusions

While more than half of participants with headache had consulted a professional HCP within the previous year, this statistic reflects demand while saying nothing of the quality of care given. Neither the investigations reported nor the medications used offer reassurance, while high persistent burden more plainly indicates these consultations fall far short of meeting need. Health policy in Nepal would do well to recognise this: the consequences otherwise are costly – in lost health, in diminished productivity, and to the national economy. Importantly, capacity appears to exist at multiple levels within the Nepalese health system. With this to build upon, structured headache services in line with international recommendations appear to be achievable in Nepal, despite the obvious difficulties. The essential requirement is educational.

## Abbreviations

AOR: Adjusted odds ratio
CI: Confidence interval
CT: Computed tomography
EEG: Electroencephalography
ENT: Ear, nose and throat
GBD: Global Burden of Disease study
HARDSHIP: Headache-Attributed Restriction, Disability, Social Handicap and Impaired Participation
HCP: Health-care provider
ICHD: International Classification of Headache Disorders
LAMI: Low- and middle-income
LTB: *Lifting The Burden*
MOH: Medication-overuse headache
MRI: Magnetic resonance imaging
NPR: Nepalese rupee
NSAID: Nonsteroidal anti-inflammatory drug
OR: Odds ratio
PHC: Primary health-care centre
pMOH: Probable MOH
PNS: Paranasal sinuses
SD: Standard deviation
SEAR: South-East Asia Region
SPSS: Statistical Package for Social Sciences
TTH: Tension-type headache
USD: United States dollar
YLD: Year of life lost to disability
